# Does Sunlight Affect the Quality for Purposes of DNA Analysis of Blood Stain Evidence Collected from Different Surfaces?

**DOI:** 10.3390/genes15070888

**Published:** 2024-07-06

**Authors:** Livia Sliskovic, Ivana Milos, Antonia Zecic, Sendi Kuret, Davorka Sutlovic

**Affiliations:** 1 Department for Forensic Sciences, University of Split, 21 000 Split, Croatia; lsliskovic@forenzika.unist.hr (L.S.); antoniazecic@gmail.com (A.Z.); 2 Department of Integrative Physiology, School of Medicine, University of Split, 21 000 Split, Croatia; ivana.banic@mefst.hr; 3 Department of Health Studies, University of Split, 21 000 Split, Croatia; sendikuret@gmail.com; 4 Department of Toxicology and Pharmacogenetics, School of Medicine, University of Split, 21 000 Split, Croatia

**Keywords:** forensic DNA analysis, crime blood stains, sunlight exposure, DNA damage

## Abstract

The aim of this study was to investigate the effect of sunlight on the degradation of DNA samples taken from blood stains from different types of surfaces. A blood sample obtained from a single male donor was placed on seven different surfaces (galvanized sheet, iron rod, newspaper, white printer paper, glass, soil, and ceramic panel). Samples were kept, during a 4-week summer period, in a room, but next to an open window. Every 7 days, 1 mm^2^ of blood sample was collected from each substrate and stored in labeled tube for later analysis. DNA was extracted with the Chelex method, amplified using AmpFISTR^TM^ Minifiler^TM^ Plus Amplification Kit, and quantified using a Quantifiler^TM^ Human DNA Quantification kit. After 7 days of sun exposure, the highest DNA concentration was determined to be from the sample from a galvanized sheet stain, followed by, in order of decreasing concentration, the ceramic panel, glass, newspaper, iron rod, and white printer paper surface. As expected, the DNA concentration from all samples decreased as the sunlight exposure time progressed. The results obtained after the amplification in the MiniFiler^TM^ system were in correlation with the DNA concentrations measured by the qPCR method for all samples, except for the glass, soil, and white printer paper samples. The obtained data show that DNA degradation is correlated to the length of sunlight exposure and to the type of surface the samples are collected from. A negative qPCR result does not mean negative PCR amplification in the STR system; therefore, both methods should be applied when analyzing forensic samples collected from trace evidence.

## 1. Introduction

Crime-scene reconstruction relies on the combined efforts of medical examiners, criminalists, and law enforcement personnel to recover physical evidence. Samples that are capable of being subjected to DNA analysis are limited to those which are of biological origin, so investigators often try to find blood stains, fresh or dried, during their examination of a crime scene, in order to possibly solve crimes using DNA profiling [1]. Since improperly handled blood evidence can weaken or destroy a potential source of facts relevant to the case, it is essential to properly document, collect, and store this type of evidence; later, it can be presented to a judge or jury many years from the time of the criminal act. Perhaps the most powerful application of DNA evidence is its ability to absolutely eliminate a person as a potential suspect in a crime [2]. Biological evidence such as blood can degrade with time and with exposure to sunlight and UV radiation, temperature, moisture, or microbial activity [3,4,5]. UV radiation is responsible for DNA damage [6,7]. The UV portion of the electromagnetic spectrum consists of three types of light: UVA (320–400 nM), UVB (290–320 nM), and UVC (100–290 nM). UVA rays make up 95% of the rays reaching Earth and UVB rays make up 5%, while UVC rays are absorbed by stratospheric oxygen and do not reach the Earth’s surface [5]. Exposure to solar UV radiation can cause a number of different types of damage in the DNA molecule. For example, UVB radiation directly produces DNA lesions through formation of DNA photoproducts [8], while the UVA and visible light energy can damage DNA indirectly via photosensitizing reactions by generating highly reactive chemical intermediates such as singlet oxygen. Singlet oxygen (^1^O_2_) is an excited state of molecular oxygen and a form of reactive oxygen species (ROS) that can induce DNA damage (DNA strand breaks and base modifications) [9,10,11].

DNA typing plays a critical role within the criminal justice system now, but one of the limiting factors with this technology is that DNA, when extracted from biological stains recovered from the crime scene, is sometimes too damaged to be tractable to analysis [12]. Potential remedies for damaged DNA are likely to be dependent upon the precise nature of the DNA damage present in any particular sample, but, unfortunately, the current knowledge of the biochemical nature and the extent of such DNA damage in dried biological stains is rudimentary [12]. Many researchers have analyzed the possibility of detecting biological samples with regard to different surfaces and different influences [13,14,15].

The aim of this study was to investigate the effect of sunlight exposure on blood stains collected from different types of surfaces during a period of 28 days. Another aim was to determine the type of surface from which, under the same conditions, we could obtain a better quality and higher quantity of extracted DNA.

## 2. Materials and Methods

### 2.1. Sample Collection

The blood sample was obtained from a single male donor, aged 25, after informed consent had been obtained. Venous blood samples were collected using EDTA vacutainers (Becton Dickinson GmbH, Franklin Lakes, NJ, USA) and stored in 1.7 mL microcentrifuge tubes at 4 °C until analysis.

### 2.2. Sample Preparation

**Figure 1:**
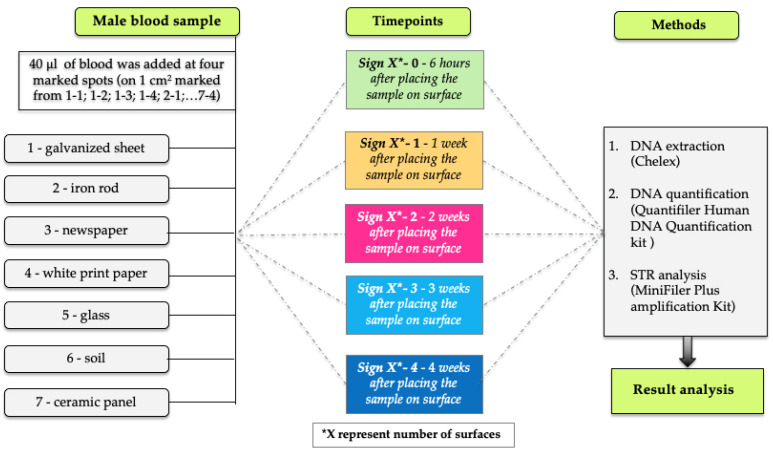
Research design. Blood samples on seven different surfaces at five timepoints. X* represents number of surfaces.

The blood sample was vortexed to ensure homogeneity. On each of seven different surfaces (galvanized sheet, iron rod, newspaper, white printer paper, glass, soil, and ceramic panel; marked from 1–7) 40 μL of blood was placed at four marked spots (marked as 1-1; 1-2; 1-3; 1-4; 2-1; …7-4). Each blood stain was spread over the surface of 1 cm^2^. The samples were placed in an indoor environment, but immediately next to an open glass surface (window) which ensured sunlight exposure for the period of four weeks during summer (dates within June and July). Research design is shown in Figure 1. The window was open during the daytime and closed at nighttime in order to allow the UVA and UVB rays to reach the samples, since it is known that standard glass filters out UVB, while the UVA rays are still transmitted [16]. In the room where the samples were placed, relative humidity and temperature were measured daily during the research period.

A negative control (Nuclease free water, Thermo Fisher Scientific Inc., Waltham, MA, USA) without sample was included with each batch of samples analyzed.

Averages of daily outdoor temperatures and humidity were determined (Table 1). Throughout all 28 days, the weather was mostly sunny, except for two partially rainy days in the second week. During those two days, the relative humidity was 74 and 79%.

Every 7 days, duplicate samples from a correspondingly marked surface spot were taken (the sample marked 1-0 was taken from surface 1 initially, 6 h after placing the sample on the surface; the 1-1 sample was taken from surface 1 after the first week; the 1-2 sample was taken from surface 1 after two weeks; the 1-3 sample was taken from surface 1 after 3 weeks; the 1-4 sample was taken from surface 1 after 4 weeks; and so through 7-4). Finally, after 28 days, 10 samples from each substrate type were analyzed; in total, 70 samples, plus the negative control (also in duplicate), were analyzed. From the paper, a 1 mm^2^ area was cut out, while from the metal, ceramic and glass surfaces, an equivalent area of sample was swept with a single wet swab technique using a sterile cotton swab (Puritan^TM^, Guilford, ME, USA) moistened with sterile water (40 μL). All samples were stored in labeled test tubes. Soil samples of 1 mm^2^ area were also transferred to selected labeled tubes.

### 2.3. DNA Extraction

Using a sterile scalpel, 1 mm^2^ of blood sample was cut from the paper samples. For swab samples, the tip of the swab was cut off and transferred to the marked tube. DNA extraction for all samples was performed using 200 μL 5% Chelex 100 solution (Bio-Rad Laboratories GmbH, Hercules, CA, USA) [17] and 3 μL proteinase K, 20 mg/mL (Roche GmbH, Basel, Switzerland). The tubes were incubated in thermomixer for 1 h at 56 °C, with low agitation (300 rpm), and subsequently boiled for 8 min at 96 °C. After centrifugation for 3 min in a microcentrifuge at 12,000 rpm, the supernatant was transferred into a new tube and was stored at −20 °C pending further analyses.

### 2.4. DNA Quantification

Extracted DNA was quantified using the Quantifiler^TM^ Human DNA Quantification kit (Applied Biosystems, Foster City, CA, USA) according to the manufacturer’s instructions. The analysis was performed on the ABI Prism 7000 Sequence Detection System (Applied Biosystems, Foster City, CA, USA) and the ABI Prism 7000 SDS software v. 1.0.1. The quantification assay was performed in a total volume of 50 µL of a reaction mix containing DNA sample, Quantifiler^TM^ human primer mix, and Quantifiler^TM^ PCR reaction mix, according to the manufacturer’s protocols [18]. The CT value was set to a default threshold of 0.20 for all reactions, and the copy of number value for unknown samples was inferred from the regression line of standard curves.

### 2.5. Autosomal STR Analysis

Amplification of DNA samples was performed with the AmpFISTR Minifiler^TM^ Plus Amplification Kit (Applied Biosystems, Foster City, CA, USA) according to the manufacturer’s instructions [19]. This kit allows simultaneous amplification of eight large autosomal STR loci (D13S317, D7S820, D2S1338, D21S11, D16S539, D18S51, CSF1PO, and FGA) and the amelogenin locus (determining the individual’s sex) and is optimized for use with degraded DNA samples. The PCR products were analyzed on ABI PRISM 310 Genetic Analyzer (Applied Biosystems, Foster City, CA, USA).

### 2.6. Statistical Analysis

The Kolmogorov–Smirnov test was used for normality checking. Due to the non-normal distribution of the data, the correlation between DNA concentration and the number of successfully amplified loci was determined using the non-parametric Spearman’s Rho Correlation test. The chi-square test was used to analyze differences in concentrations with respect to the number of exposure days. Statistical analysis was performed using the Statistical Package Software for Social Science, version 28 (SPSS Inc., Chicago, IL, USA).

## 3. Results

The negative control showed no DNA profile, indicating that no contamination had occurred. The experiment’s results showed (Table 2) a decrease in the concentration of DNA extracted from blood stains, as quantified by qPCR, as the period of sunlight exposure increased. The result represents the mean values of the two parallel samples taken from each spot. In Supplementary Figures S1A–S4A, the results of the amplification plot of the DNA, as detected by qPCR with FAM- and VIC-labeled probes, are shown. Results of the STR amplification MiniFiler^TM^ system are shown in Supplementary Figures S1B–S4B.

**Figure 2:**
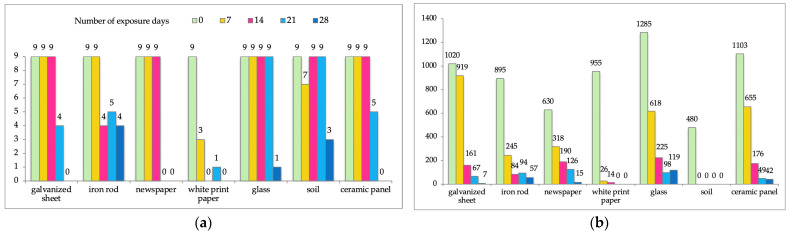
Changes according to the number of exposure days (0, 7, 14, 21, and 28), taken from different surface samples (X-axis): (**a**) changes in number of successfully amplified loci (Y-axis) and (**b**) changes in DNA concentrations (Y-axis shows the DNA concentration in pg/μL).

Decreasing concentrations of DNA consistently revealed higher CT numbers. The highest DNA concentration was determined to be that from a galvanized sheet stain after 7 days (919 pg/μL; CT values 27.86), and the lowest concentration was from a white-paper stain after 21 days (0.33 pg/μL; CT values 38.52). In all samples taken from the soil surface, and in one sample from a white-paper stain after 28 days, the presence of DNA was not determined. After 7 days of sun exposure, the highest DNA concentration was determined to be that from a galvanized sheet stain, and, subsequently, in order of decreasing concentration, from ceramic panel, glass, newspaper, iron rod, and white printer paper surfaces. Concentrations were 919, 655, 618, 318, 245, and 26 pg/μL, respectively. After 14 and 21 days of sunlight exposure, DNA concentrations from all surfaces decreased in different ways (Figure 2). The results obtained after amplification in the MiniFiler^TM^ system were in good correlation with the DNA concentration measured by the qPCR method for all samples, except for glass and soil samples; the *r_s_* factors were 0.353 and 0.395, respectively (Table 2 and Figure 2).

**Figure 3:**
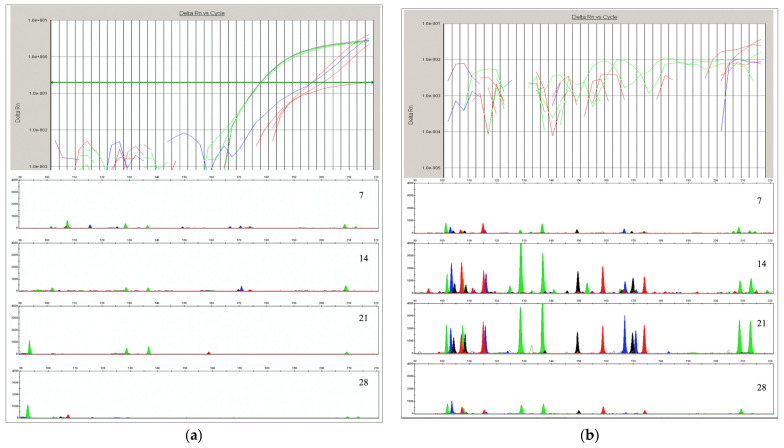
The results of the amplification plot of the DNA, as detected by qPCR with FAM- and VIC-labeled probes (**above**), and the STR amplification MiniFiler^TM^ system (**below**), as extracted from blood stains from (**a**) white printer paper and (**b**) soil surfaces, according to the number of sunny days (7, 14, 21, and 28).

For samples collected from blood stains deposited on white paper and soil, the results of the DNA amplification plot, as detected by qPCR with FAM- and VIC-labeled probes, showed a discrepancy with the results of the STR amplification of the MiniFiler^TM^ system. It was observed that fewer STR loci were amplified from blood stains from white paper, as compared to the blood stains from a soil sample, although the quantitative result was significantly better (Figure 3).

**Figure 4:**
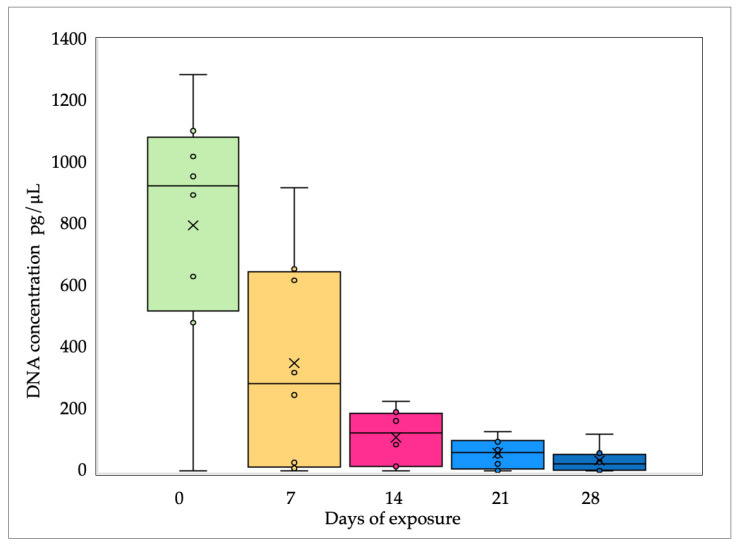
Changes in DNA concentration (Y-axis—DNA in pg/μL) according to the number of exposure days (X-axis—0, 7, 14, 21, and 28 days), as determined in samples from different surfaces.

Finally, when comparing the results for all substrates with regard to different surfaces, the concentration of DNA significantly decreases with the number of days of exposure to the sun. Thus, the median concentration decreased from 925 pg/μL after the first day to 281.5 pg/μL after 7 days, to 122.5 pg/μL after 14 days, to 58 pg/μL after 21 days, and, finally, to 21.5 pg/μL after 28 days. The greatest decline (about 70%) was observed in the first 7 days (Figure 4), while afterwards, that percentage was lower. The chi-square test shows a statistically significant difference (*p* < 0.05) between the results of DNA concentrations for samples obtained from different substrates, although they were taken in the same interval (every 7 days).

## 4. Discussion

Among the forms of biological evidence found at the crime scene, blood is the most common. DNA typing of biological evidence has revolutionized criminal investigations, aiding in linking perpetrators to the victim as well as the crime scene [20]; DNA persistence and transfer are crucial factors in evaluating a crime scene. Determining how long DNA can remain at a specific location is as important as understanding how it was transferred there [21]. Research on DNA persistence is still very limited and has primarily been carried out in laboratory settings [22,23,24]. Especially, the influence of weather on DNA stability is a very rare topic.

Previous studies have shown that environmental factors such as temperature, humidity, sunlight exposure, and substrate type can influence DNA stability and degradation in biological samples [7,25]. In this study we wanted to investigate the effects of some of those factors, i.e., sunlight exposure and type of surface, on the quality of DNA extracted from blood stains. In order to protect samples from environmental conditions that could potentially destroy them (e.g., wind and rain), we decided to place the samples in a room, but immediately next to an open window, ensuring exposure to both UV-A and UV-B radiation. We believe that the temperature and humidity did not significantly contribute to the DNA degradation in this study. In other words, the indoor temperature and humidity were relatively stable during the 4-week period, and did not fluctuate much. During that period, the weekly average or daily temperature was 25.1–30.3 °C (33 °C was the highest temperature measured, occurring in the third week of the experiment). It has been reported that high temperatures (above 55 °C) contribute to DNA degradation [26,27].

Furthermore, the recorded humidity values were moderate, since the average of daily relative humidity was 48.9–54.5% during the 4 weeks of the experiment. There were only two rainy days during which humidity levels were high, with levels of 74% and 79%, respectively. Chen et al. have observed that an average humidity level of 64% during a summer period did not contribute significantly to DNA preservation [28].

### 4.1. DNA Quantification

With the increased sensitivity and sophistication of DNA detection methods seen over the past 20 years, it has become possible to obtain DNA profiles from samples with very low DNA concentrations. A reliable and highly sensitive DNA quantification system is necessary to ensure an optimum use of the limited material available.

DNA concentrations were successfully determined for all samples extracted from galvanized steel, iron rods, newspaper, glass, and ceramics. As expected, the DNA concentrations from all samples decreased as sunlight exposure time progressed (Figure 1). In all samples collected from the soil surface, DNA could not be quantified using the qPCR technique. However, corresponding DNA samples, when taken after 14 and 21 days, were successfully amplified in the MiniFiler^TM^ amplification system. On the other hand, DNA concentrations of 26 and 14 pg/μL were determined to be present in the white printer paper samples after 7 and 14 days, as determined by qPCR, but neither of the samples were successfully amplified in the MiniFiler^TM^ amplification system. In the samples taken from the white printer paper after 28 days, DNA was not detected. In seeking to explain the low DNA concentrations in those samples, aside from sunlight exposure, another contributing factor could be the chemicals that are used in the paper manufacturing process. The bleaching of paper is performed using products based on chlorine, chlorine dioxide, or nonchlorinated bleaching agents such as oxygen and peroxide [29].

Bleach causes oxidative damage to nucleic acid and prevents it from being reamplified in subsequent PCR reactions [30]. Alaeddini et al. indicated in their review that oxidative damage mostly includes modifications of sugar residues, conversion of cytosine and thymine to hydantoins, and removal of bases and cross-linkages, which could block PCR amplification [31].

Our previous studies have shown that soil samples can inhibit qPCR and PCR reactions [32,33]. The extraction of total DNA from soil always results in co-extraction of some other soil components, mainly humic acid (HA) and other humic substances, which adversely affect DNA-detecting processes [34,35,36]. Considering those findings, as well as the contribution of sunlight exposure, the possibility of the inability to detect DNA in blood stains collected from that surface was expected.

Samples collected from newspaper and an iron rod instantly had a lower DNA concentration after 7 days. Newspaper ink can consist of components such as lead [37], while the rod contained iron. Previous studies demonstrated that the presence of heavy metals such as lead and iron can also inhibit PCR reactions [38,39]. Samples taken from galvanized sheet, glass, and ceramic panel showed the greatest concentrations of DNA. Those concentrations were significantly reduced after 7 days, but did not drop below zero after 28 days, even though their quantities were not sufficient for successful amplification. In the first part of their study on trace DNA and its persistence on various surfaces, Arsenault et al. assessed the capacity of three different DNA types (cell free DNA (cfDNA), cellular DNA, and a mixture) to persist on seven different metals, including aluminum, brass, copper, galvanized steel, lead, mild steel, and tin. Their results showed that DNA persistence was the longest on lead surfaces, followed by galvanized steel, aluminum, mild steel, and tin, while brass and copper had the lowest DNA persistence rates [40]. The results of this study do not fully support the results of our study, in which the success of DNA analysis of samples obtained from a newspaper surface was attributed to the presence of lead as known PCR inhibitor. Since it involved a different medium with different porosity, it was entirely impossible to determine the influence of lead on the success of the analysis.

### 4.2. Autosomal STR Analysis

Human identification is the primary goal of most forensic DNA casework, and STR genotyping is typically the analytical method of first choice because of its high power of discrimination [41].

Different degrees of degradation appear to have different negative impacts on the amplification process, especially for STR systems with large amplicons. As expected, decreasing the concentration of DNA significantly reduced the success rates of PCR amplifications. Methods which need only small amplicon sizes to detect DNA markers are a better choice for typing degraded DNA [42]. Nevertheless, amplification with the MiniFiler^TM^ Amplification system did not give a complete profile on nine loci in all analyzed samples. This could be due to insufficient DNA concentrations (values ranged between 0.33 and 1285 pg/μL). A diploid human cell contains about 6.6 pg/μL of genomic DNA. A template DNA concentration <100 pg/μL genomic DNA (about 15–17 diploid copies of nuclear DNA markers such as autosomal STRs) is considered to be a low copy number [31,43]. In this study, in twelve of twenty-eight analyzed samples, the DNA quantity was less than 100 pg/μL, while in five samples, DNA was not detected. This means that 17 of 28 samples did not have enough copies of nuclear DNA. After seven sunny days, DNA from all samples, except those from white printer paper and soil surfaces, was successfully amplified at all loci. The range of DNA concentration in tested samples was between 245 and 919 pg/μL. After 14 days, the DNA concentration was between 161 and 225 pg/μL, and samples from all examined surfaces, except those from white printer paper and iron rod surfaces, were successfully amplified at all loci. However, after 21 days, DNA was successfully amplified from only two samples, which were collected from the glass and soil surfaces. The DNA concentration from the glass surface sample was 98 pg/μL, while the DNA concentration from the soil surface could not been detected using qPCR method. None of the DNA samples taken after 28 days were successfully amplified in any loci. Figure 2a shows the success of amplification rate according to the number of sunny days.

The results of our study are consistent with the results of the study conducted by Alketbi and Goodwin [44]. They analyzed the success of the DNA analysis of touch DNA in relation to the type of surface. According to their results, which were similar to ours, the least successful analysis was from samples taken from the surface of white photocopier paper, while the most successful DNA analysis was from samples obtained from a glass surface. They also emphasized that the sampling method impacted the results. Furthermore, the sampling method and the success of the analysis of touch DNA samples from glass surfaces were specifically investigated by Schulte et al. [45]. They concluded that a sampling method using detergent-based solutions significantly increased DNA recovery efficiency.

A recent study by Ricci et al., has shown that successful PCR amplification is possible, even with very low concentrations of template DNA. The concentration threshold for PCR amplification was 0.003 ng/μL, with a maximum reaction volume of 15 μL [46]. STR multiplexes usually work optimally with 0.5–1 ng template DNA with 28–32 amplification cycles [19,31]. One nanogram of DNA is approximately equal to 660 copies of genomic DNA. An increased incidence of PCR failures was observed when the starting DNA concentration was less than 60 copies [31,47]. Except for DNA samples taken from soil, in which DNA was not detected, the DNA sample taken from the glass surface after 21 days had the lowest DNA concentration (98 pg/μL, approximately 60 copies), and yet it was successfully amplified at all loci. This could be due to type of glass surface and the absence of PCR inhibitors. But after 28 days, the DNA concentration was 119 pg/μL and the DNA was amplified in just 1 locus. We believe that in that sample, as well as in all other analyzed samples, exposure to sunlight had a major impact in its contribution to the DNA degradation. Thacker et al., as well as other authors, have shown that UV light causes the dropout of alleles and incomplete DNA profiles, even after a short time exposure (2 min) [3,12]. In another research project, conducted by Rahi et al., whole human blood samples from a single male donor were exposed to UVA, UVB, UVC, and solar radiation at 20 min intervals up to 120 min and allele frequencies of 16 STR markers were monitored. The results showed that UVA and solar radiation did not cause any noticeable DNA damage within this period of time, while UVB and UVC caused some STR markers either to not amplify or to amplify with low RFUs [9]. Even though it is known that other environmental factors, such as temperature and humidity, also play a role in DNA persistence, one study showed that normal climatic conditions are not critical for the longtime survival of DNA in untreated blood stains [48]. Despite the fact that the availability of water is important for processes that degrade DNA, no significant differences were observed in the stability of DNA at 50%, 80%, or 93% relative humidity, either at room temperature or at 35 °C. Similar findings were reported in a study by Hall et al. [12]. Given that, in our study, the relative humidity and temperature values were in accordance with those values, we can conclude that sunlight exposure (UV radiation) was the dominant environmental factor affecting DNA degradation/quality. Overall, the DNA concentrations in blood samples collected from all surfaces decreased as the period of sunlight exposure increased.

Another factor that could have influenced the results of our study is the type of surface on which the blood samples were placed, which can be attributed to the presence of various PCR inhibitors, as we have explained earlier.

### 4.3. Study Limitations

There are several limitations of this study that should be addressed in future research. The level of UV radiation was not measured, which would contribute to characterization of sunlight exposure as an environmental factor influencing DNA stability and degradation. Research could also be conducted on additional substrate types often found at crime scenes (e.g., fabric) as well as different biological samples (e.g., saliva, and semen) in order to gain better insight into variations in DNA preservation.

To further understand DNA preservation, the influence of additional environmental factors such as humidity and temperature should be investigated. This could involve setting up experimental environments with elevated temperature and humidity levels. Also, by adding new variables, multivariate analysis could be performed.

## 5. Conclusions

The results of this study could assist law enforcement agencies during investigations and in court in assessing the likelihood of successful DNA typing and informing decisions regarding the relevance of DNA recovered from crime scenes. Our findings highlight the importance of early sampling of blood traces from the crime scene. Any delay represents a possibility of incomplete determination of DNA profiles. This is particularly important during the summer months and periods of more intense sunlight exposure.

Overall, the DNA concentrations in blood samples collected from all surfaces decreased as the period of sunlight exposure increased. The results obtained after the amplification in the MiniFiler^TM^ system were in correlation with the DNA concentrations determined for all samples, except for the glass, soil, and white printer paper samples. The number of amplified STR loci varied among the samples, depending on the type of substrate and the time of exposure. After 28 days, it was not possible to amplify all 9 STR loci in any tested sample, regardless of the surface type.

Comparing the surface types, it is evident that the best DNA analysis results were obtained from blood stain samples collected from glass, ceramics, galvanized steel, iron rods, and newsprint, respectively. Those variations can be attributed to the presence of different PCR inhibitors.

In conclusion, sunlight exposure (UV radiation) and surface type are environmental factors affecting DNA quality and quantity. Future studies should investigate the impact of additional factors such as humidity and temperature, as well as other types of surfaces (e.g., fabric) commonly encountered at crime scenes.

## Figures and Tables

**Table 1:** The indoor conditions data (temperature and humidity) during the experimental period.

Sunlight Days	Average of Daily (Day/Night) Indoor Temperatures (°C)	Average of Daily Relative Humidity (%)
1–7	25.1	48.9
8–14	27.3	59.0
15–21	30.3	50.6
22–28	27.4	54.5

**Table 2:** The results associated with different DNA concentrations and the corresponding values of amplification cycles, Ct value (average value of two parallel analyses), and number of loci, as successfully amplified in MiniFilier^TM^ Amplification system, and sorted according to the tested surface and the number of sunny days. (The sample numbers are composed of two numbers: the first indicates the number of surfaces, and the second number indicates the week of sampling).

Sample Number	Type of Surface	Number of Days of Sunlight	Ct	DNA Concentration (pg/μL)	Number of Successfully Amplified Loci (Including Amelogenin)	Spearman’s Rho Correlation Coefficient *
**1-0**	1-Galvanized sheet	0	27.10	1020	9/9	*r_s_* = 0.894 *p* < 0.05
**1-1**		7	27.86	919	9/9	
**1-2**		14	30.11	161	9/9	
**1-3**		21	31.16	67	4/9	
**1-4**		28	34.23	7	0/9	
**2-0**	2-Iron rod	0	27.45	895	9/9	*r_s_* = 0.949 *p* < 0.05
**2-1**		7	29.82	245	9/9	
**2-2**		14	30.47	84	4/9	
**2-3**		21	30.33	94	5/9	
**2-4**		28	30.93	57	4/9	
**3-0**	3-Newspaper	0	28.29	630	9/9	*r_s_* = 0.866 *p* = 0.058
**3-1**		7	29.20	318	9/9	
**3-2**		14	29.82	190	9/9	
**3-3**		21	30.37	126	0/9	
**3-4**		28	33.01	15	0/9	
**4-0**	4-White printer paper (80 g)	0	27.65	955	9/9	*r_s_* = 0.821 *p* = 0.089
**4-1**		7	32.52	26	3/9	
**4-2**		14	33.49	14	0/9	
**4-3**		21	38.52	0.33	1/9	
**4-4**		28	Undetected **	Undetected	0/9	
**5-0**	5-Glass	0	27.05	1285	9/9	*r_s_* = 0.353 *p* = 0.559
**5-1**		7	28.38	618	9/9	
**5-2**		14	29.66	225	9/9	
**5-3**		21	30.67	98	9/9	
**5-4**		28	30.61	119	1/9	
**6-0**	6-Soil	0	29.20	480	9/9	*r_s_* = 0.395 *p* = 0.510
**6-1**		7	Undetected	Undetected	7/9	
**6-2**		14	Undetected	Undetected	9/9	
**6-3**		21	Undetected	Undetected	9/9	
**6-4**		28	Undetected	Undetected	3/9	
**7-0**	7-Ceramic panel	0	27.15	1103	9/9	*r_s_* = 0.894 *p* < 0.05
**7-1**		7	28.31	655	9/9	
**7-2**		14	29.91	176	9/9	
**7-3**		21	31.51	49	5/9	
**7-4**		28	31.70	42	0/9	

* Spearman’s Rho Correlation Coefficient: relationship between DNA concentration and number of successfully amplified loci. ** Undetected = concentration below threshold.

## Data Availability

The original contributions presented in the study are included in the article, further inquiries can be directed to the corresponding author.

## Supplementary Materials

The following supporting information can be downloaded at: https://www.mdpi.com/article/10.3390/genes15070888/s1. Figure S1: The results from analyzed surface samples (galvanized sheet; iron rod; newspaper; white printer paper; glass; soil; and ceramic panel; respectively, 1 to 7), taken after 7 days: (A) amplification plot of the DNA, as detected by QRT PCR with FAM- and VIC-labeled probes; and (B) results of the STR amplification plot in the MiniFiler^TM^ system. Figure S2: The results from analyzed surface samples (galvanized sheet; iron rod; newspaper; white printer paper; glass; soil; and ceramic panel; respectively, 1 to 7), taken after 14 days: (A) amplification plot of the DNA, as detected by QRT PCR with FAM- and VIC-labeled probes; and (B) results of the STR amplification plot in the MiniFiler^TM^ system. Figure S3: The results from analyzed surface samples (galvanized sheet; iron rod; newspaper; white printer paper; glass; soil; and ceramic panel; respectively, 1 to 7) taken after 21 days: (A) amplification plot of the DNA, detected by QRT PCR with FAM- and VIC-labeled probes; and (B) results of the STR amplification plot in the MiniFiler^TM^ system. Figure S4: The results from analyzed surface samples (galvanized sheet; iron rod; newspaper; white printer paper; glass; soil; and ceramic panel; respectively, 1 to 7), taken after 28 days: (A) amplification plot of the DNA, as detected by QRT PCR with FAM- and VIC-labeled probes; and (B) results of the STR amplification plot in the MiniFiler^TM^ system.

## References

[1] Krawczak M., Schmidtke J. (1994). DNA Fingerprinting.

[2] Crime Scene Investigator Network Wildomar (CA): Crime Scene Resources Inc; c2000–2015. Collection and Preservation of Blood Evidence from Crime Scenes; [cited 10 June 2014]; [about 9 Screens]. http://www.crime-scene-investigator.net/blood.html.

[3] Thacker C.R., Oguzturun C., Ball K.M., Syndercombe Court D. (2006). An investigation into methods to produce artificially degraded DNA. Int. Congr. Ser..

[4] Goodsell D.S. (2001). The molecular perspective: Ultraviolet light and pyrimidine dimers. Oncologist.

[5] Hall A., Sims L.M., Ballantyne J. (2014). Assessment of DNA damage induced by terrestrial UV irradiation of dried bloodstains: Forensic implications. Forensic Sci. Int. Genet..

[6] Maverakis E., Miyamura Y., Bowen M.P., Correa G., Ono Y., Goodarzi H. (2010). Light, including ultraviolet. J. Autoimmun..

[7] Khorwal D., Mathur G.K., Ahmed U., Daga S.S. (2024). Environmental Factors Affecting the Concentration of DNA in Blood and Saliva Stains: A Review. J. Forensic Sci. Res..

[8] El-Yazbi A.F., Loppnow G.R. (2014). Detecting UV-induced nucleic-acid damage. TrAC Trends Anal. Chem..

[9] Rahi G.S., Adams J.L., Yuan J., Devone D.-J., Lodhi K.M. (2021). Whole human blood DNA degradation associated with artificial ultraviolet and solar radiations as a function of exposure time. Forensic Sci. Int..

[10] Hrycay E.G., Bandiera S.M. (2015). Involvement of Cytochrome P450 in Reactive Oxygen Species Formation and Cancer. Adv. Pharmacol..

[11] Cadet J. (2000). Singlet oxygen induces oxidation of cellular DNA. J. Biol. Chem..

[12] Hall A., Ballantyne J. (2004). Characterization of UVC-induced DNA damage in bloodstains: Forensic implications. Anal. Bioanal. Chem..

[13] Medina-Paz F., Kuba B., Kryvorutsky E., Roca G., Zapico S.C. (2024). Assessment of Blood and Semen Detection and DNA Collection from Swabs up to Three Months after Deposition on Five Different Cloth Materials. Int. J. Mol. Sci..

[14] Jäger R. (2022). New Perspectives for Whole Genome Amplification in Forensic STR Analysis. Int. J. Mol. Sci..

[15] Chierto E., Aneli S., Nocco N., Riem A., Onofri M., Carnevali E., Robino C. (2024). Assessing DNA Degradation through Differential Amplification Efficiency of Total Human and Human Male DNA in a Forensic qPCR Assay. Genes.

[16] Tuchinda C., Srivannaboon S., Lim H.W. (2006). Photoprotection by window glass, automobile glass, and sunglasses. J. Am. Acad. Dermatol..

[17] Walsh P.S., Metzger D.A., Higuchi R. (1991). Chelex 100 as a medium for simple extraction of DNA for PCR-based typing from forensic material. Biotechniques.

[18] Applied Biosystems Quantifiler^®^ Human DNA Quantification Kit and Quantifiler^®^ Y Human Male DNA Quantification Kit: User Manual. Foster City (CA): Life Technologies Corporation; 2012 March [cited 10 June 2014], 216p. http://www3.appliedbiosystems.com/cms/groups/applied_markets_support/documents/generaldocuments/cms_041395.pdf.

[19] Mulero J.J., Chang C.W., Lagace R.E., Wang D.Y., Bas J.L., McMahon T.P., Hennessy L.K. (2008). Development and validation of the AmpFISTR MiniFiler PCR amplification kit: A MiniSTR multiplex for the analysis of degraded and/or PCR inhibited DNA. J. Forensic Sci..

[20] Shrivastava P., Kumawat R.K., Kushwaha P., Rana M., Dash H.R., Shrivastava P., Lorente J.A. (2022). Biological Sources of DNA: The Target Materials for Forensic DNA Typing. Handbook of DNA Profiling.

[21] Poetsch M., Markwerth P., Konrad H., Bajanowski T., Helmus J. (2022). About the influence of environmental factors on the persistence of DNA—A long-term study. Int. J. Leg. Med..

[22] Raymond J.J., Walsh S.J., van Oorschot R.A., Gunn P.R., Evans L., Roux C. (2008). Assessing trace DNA evidence from a residential burglary: Abundance, transfer and persistence. Forensic Sci. Int. Genet. Suppl. Ser..

[23] Poetsch M., Pfeifer M., Konrad H., Bajanowski T., Helmus J. (2017). Impact of several wearers on the persistence of DNA on clothes—A study with experimental scenarios. Int. J. Leg. Med..

[24] Szkuta B., Ballantyne K.N., van Oorschot R.A.H. (2017). Transfer and persistence of DNA on the hands and the influence of activities performed. Forensic Sci. Int. Genet..

[25] Lee L.Y.C., Wong H.Y., Lee J.Y., Waffa Z.B.M., Aw Z.Q., Fauzi S.N.A.B.M., Hoe S.Y., Lim M.L., Syn C.K.C. (2019). Persistence of DNA in the Singapore context. Int. J. Leg. Med..

[26] Abdulla J.M., Gomaa R., Attalla S.M., Nader L.M. (2021). Investigation of DNA degradation in forensic blood samples after exposure to different environmental condi-tions. Int. J. Med. Toxicol. Leg. Med..

[27] Abdel Hady R.H., Thabet H.Z., Ebrahem N.E., Yassa H.A. (2021). Thermal Effects on DNA Degradation in Blood and Seminal Stains: Forensic View. Acad. Forensic Pathol..

[28] Chen C., Pistono A., Ryan S., Szkuta B., Meakin G.E. (2019). The effect of climatic simulations on DNA persistence on glass, cotton and polyester. Forensic Sci. Int. Genet. Suppl. Ser..

[29] Wikipedia San Francisco: Wikipedia, The Free Encyclopedia; c2001-2015. Bleaching of Wood Pulp; [Updated 2014 November 23; Cited 2014 August 26]; [about 6 Screens]. http://en.wikipedia.org/wiki/Bleaching_of_wood_pulp#Chlorine_dioxide.

[30] Hayatsu H., Pan S.K., Ukita T. (1971). Reaction of sodium hypochlorite with nucleic acids and their constituents. Chem. Pharm. Bull..

[31] Alaeddini R., Walsh S.J., Abbas A. (2010). Forensic implications of genetic analyses from degraded DNA—A review. Forensic Sci. Int. Genet..

[32] Sutlovic D., Definis-Gojanovic M., Andjelinovic S., Gugic D., Primorac D. (2005). Taq polymerase reverses inhibition of quantitative real time polymerase chain reaction by humic acid. Croat. Med. J..

[33] Sutlovic D., Gamulin S., Definis-Gojanovic M., Gugic D., Andjelinovic S. (2008). Interaction of humic acids with human DNA: Proposed mechanisms and kinetics. Electrophoresis.

[34] Zhou J., Bruns M.A., Tiedje J.M. (1996). DNA recovery from soils of diverse composition. Appl. Environ. Microbiol..

[35] Tebbe C.C., Vahjen W. (1993). Interference of humic acids and DNA exreacted directly from soil in detection and transformation of recombinant DNA from bacteria and yeast. Appl. Environ. Microbiol..

[36] Tsai Y.L., Olson B.H. (1992). Rapid method for separation of bacterial DNA from humic substances in sedimentes for polymerase chain reaction. Appl. Environ. Microbiol..

[37] Jadhav S., Swaroop S.S., Sankhla M.S., Rajeev K. (2021). Health Risks of Newspaper Ink when Used as Food Packaging Material. Lett. Appl. NanoBioScience.

[38] Sutlovic D., Andjelinovic S., Drmic I., Definis-Gojanovic M., Primorac D. Heavy Metals from Mass Graves Bones and Identification by Genomic DNA. Final Program and Abstracts. 3rd European—American Intensive Course in Forensic Genetics and Mayo Clinic Course in Advanced Molecular and Cellular Medicine; 2003 September 1–September 5; Zagreb, Croatia. Zagreb: Studio Hrg; 2003 September [cited 2014 August 10]. 145p. http://isabs.hr/publications/3rd_conference_Book_of_Abstracts.pdf.

[39] Dalecka B., Mezule L. (2018). Study of potential PCR inhibitors in drinking water for Escherichia coli identification. Agron. Res..

[40] Arsenault H., Kuffel A., Daeid N.N., Gray A. (2024). Trace DNA and its persistence on various surfaces: A long term study investigating the influence of surface type and environmental conditions—Part one, metals. Forensic Sci. Int. Genet..

[41] Alonso A., Andjelinovic S., Martin P., Sutlovic D., Erceg I., Huffine E., Fernández de Simón L., Albarrán C., Definis-Gojanovic M., Fernández-Rodriguez A. (2001). DNA typing from skeletal remains: Evaluation of multiplex and megaplex STR systems on DNA isolated from bone and teeth samples. Croat. Med. J..

[42] Senge T., Madea B., Junge A., Rothschild M.A., Schneider P.M. (2011). STRs, mini STRs and SNPs—A comparative study for typing degraded DNA. Leg. Med..

[43] Butler J.M. (2005). Forensic DNA Typing: Biology, Technology, and Genetics of STR Markers.

[44] Alketbi S.K., Goodwin W. (2019). The effect of surface type, collection and extraction methods on touch DNA. Forensic Sci. Int. Genet. Suppl. Ser..

[45] Schulte J., Rittiner N., Kron S., Seiberle I., Schulz I. (2023). Collecting touch DNA from glass surfaces using different sampling solutions and volumes: Immediate and storage effects on genetic STR analysis. J. Forensic Sci..

[46] Ricci U., Nutini A.L., Gerundino F., Boschi B., Pelo E. (2019). The best possible result from the minimum available. Forensic Sci. Int. Genet. Suppl. Ser..

[47] Alonso A., Martin P., Albarran C., Garcia P., Primorac D., Garcia O.L., Fernandez de Simon L., Garcia-Hirschfeld J., Sancho M., Fernandez-Piqueras J. (2003). Specific quantification of human genomes from low copy number DNA samples in forensic and ancient DNA studies. Croat. Med. J..

[48] Dissing J., Søndervang A., Lund S. (2010). Exploring the limits for the survival of DNA in blood stains. J. Forensic Leg. Med..

