# Supplementary material for: Does Sunlight Affect the Quality for Purposes of DNA Analysis of Blood Stain Evidence Collected from Different Surfaces?
Source: Genes (Basel). 2024 Jul 6;15(7):888. doi: 10.3390/genes15070888 (PMC11276042; doi:10.3390/genes15070888)
Supplement: Supplementary file 1 [file genes-15-00888-s001.zip › genes-3077617-supplementary.pdf]

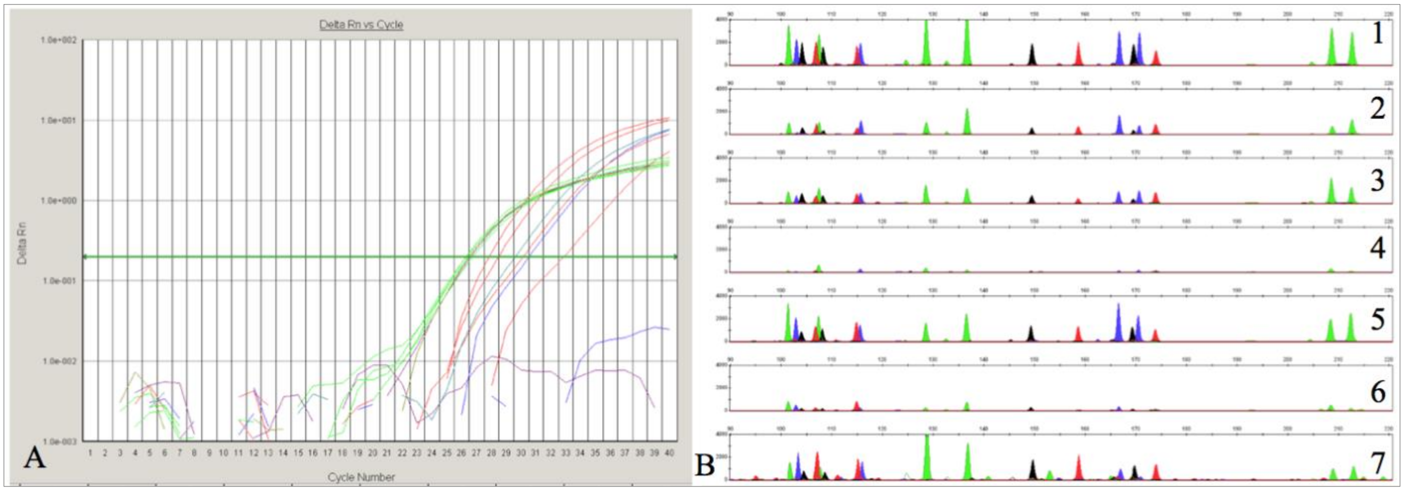

Supplementary Figure S1. The results from analyzed surface samples (galvanized sheet; iron rod; newspaper; white print paper; glass; soil and ceramic panel, respectively 1-7) taken after 7 days. (A) amplification plot of the DNA, detected by qPCR with FAM- and VIC-labeled probes; (B) results of the STR amplification plot in the MiniFiler™ system.

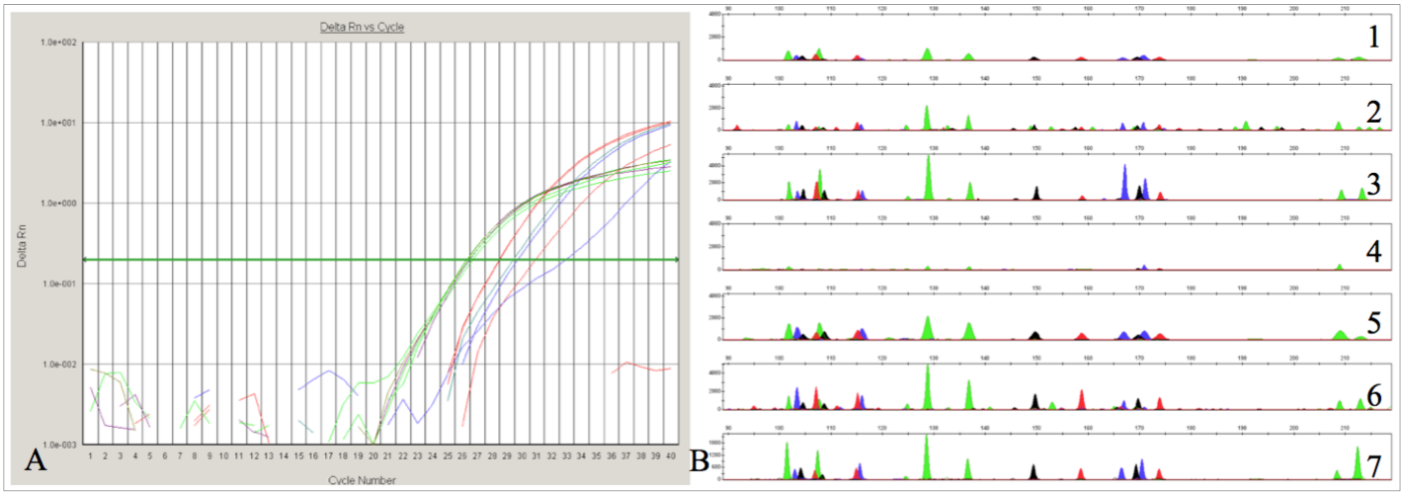

Supplementary Figure S2. The results from analyzed surface samples (galvanized sheet; iron rod; newspaper; white print paper; glass; soil and ceramic panel, respectively 1-7) taken after 14 days. (A) amplification plot of the DNA, detected by qPCR with FAM- and VIC-labeled probes; (B) results of the STR amplification plot in the MiniFiler™ system.

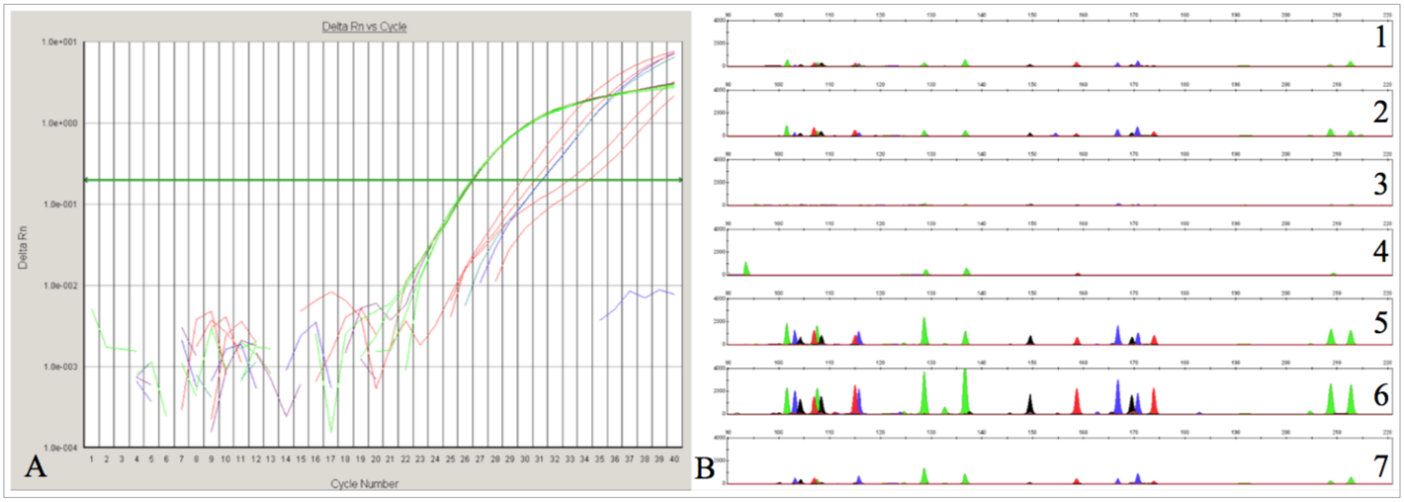

Supplementary Figure S3. The results from analyzed surface samples (galvanized sheet; iron rod; newspaper; white print paper; glass; soil and ceramic panel, respectively 1-7) taken after 21 days. (A) amplification plot of the DNA, detected by qPCR with FAM- and VIC-labeled probes; (B) results of the STR amplification plot in the MiniFiler™ system.

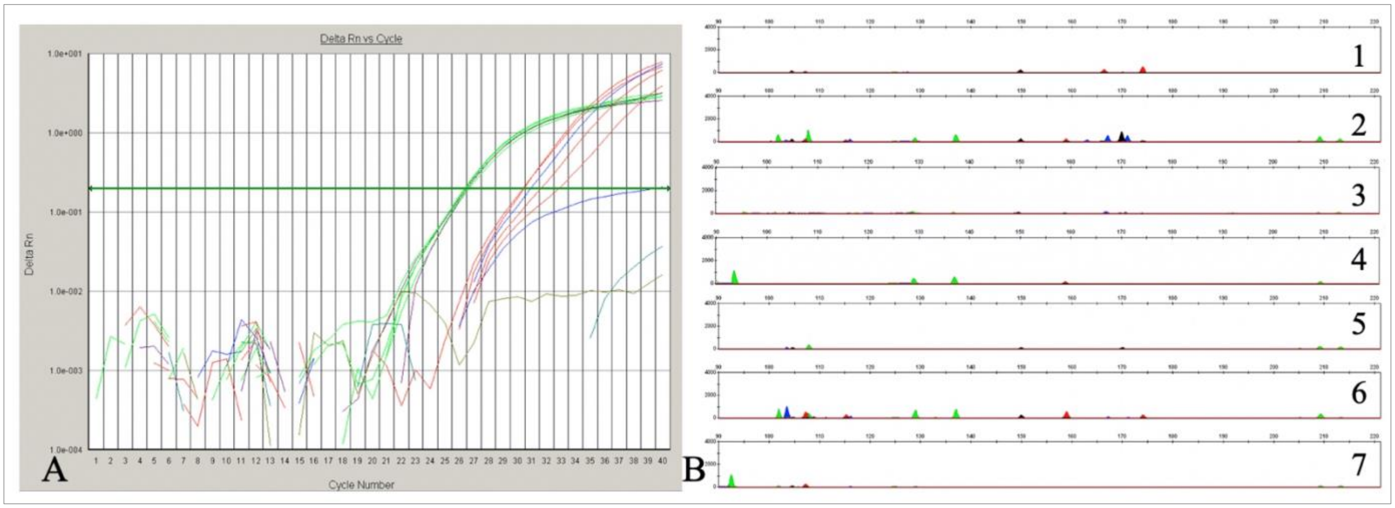

Supplementary Figure S4. The results from analyzed surface samples (galvanized sheet; iron rod; newspaper; white print paper; glass; soil and ceramic panel, respectively 1-7) taken after 28 days. (A) amplification plot of the DNA, detected by qPCR with FAM- and VIC-labeled probes; (B) results of the STR amplification plot in the MiniFiler™ system.
